# Odontomes

**Published:** 1901-11-15

**Authors:** Thomas L. Gilmer


DENTAL REGISTER.

Vol. LV. November 15, 1901. No. 11.

### ODONTOMES.

BY THOMAS L. GILMER, M.D., D.D.S.

Synopsis of a paper read at the meeting of the National Dental Association, August 8, 1901, in Milwaukee, Wis.

Odontomes are excessive growths of tooth tissues in heterogeneous mass, springing from abnormal teeth. They are exceedingly rare and have not been studied to any considerable extent. Some attempt has been made to classify them. Broca called them odontome embryoplastiques, coronaries and radicularis. Sutton classified them according to the tissues from which they took their origin: epithelial odontome from enamel organ; follicular and fibrous odontomes from the tooth follicle; cement, from cement organ and compound follicular from both, radiculose from the papilla, and composite from the whole germ.

We should not class as an odontome a tooth having an excessively developed crown or root, or a tooth having a tumor on the side of its root, or one having an enamel drop on the side of its root. A tooth more or less perfectly developed which is incisted in a thickened follicle is not an odontome, nor a tooth which is incased in a calcified bony wall. Multiocular cysts of the jaws or excementosis of the root, or impacted or unerupted supernumeraries can not be properly classed as odontomes. In other words a tumor caused by a tooth is a very different thing from a tumor composed of tooth tissues however well marked or heterogeneously the structure may be arranged.

A tumor caused by a tooth may not have any tooth structure.

Growths composed of nests of aberrant teeth or forms included in follicular cysts are questionable odontomes. They are usually known as dentigerous cysts. Sutton classes them as compound follicular odontomes.

A mass of imperfectly formed and diminutive teeth held together by an imperfect cement might be called another class of odontomes.

The composite odontome of Sutton is made up of a mixture of more or less enamel and dentine, with no surface indications of tooth forms, but microscopic sections show the dentine and enamel in true relation. These three kinds do not arise directly from the germ of the normal tooth, but are doubtless developed from some part of the tooth germs of the normal set, which has segregated in an interrupted development of the normal set. We have no definite knowledge of course as to the manner of their development, our conclusions are logical deductions or speculative. Dean in his work on the dental follicle, says they might arise from the unatrophied portion of the cord. Dr. Black thinks they may arise from the extra buds occasionally thrown out.

Dermoids in some parts of the body are caused by the inclusion of epithelial tissue in the folds of the mesoblastic layers in foetal life. These cysts may have a similar origin, but they can not be called truly dentigerous cysts.

Our literature does not record more than twelve or thirteen specimens. They may be found in either jaw, usually in the distal portions. They develop in early life and are usually observed before twenty-five years. They are usually encysted in a follicular wall. In some cases the particles of the cyst are shaped like teeth, and in others they are without definite form. If like teeth they are diminutive, and of the cuspid or bicuspid form.

A microscopic section of an odontome, reported to the Illinois Dental Society in 1879, shows all the tissues of a normally developed tooth, but in a state of confusion. The arrangement of the tissues is peculiar; it is as though a great number ot deminutive teeth were packed together and the interstices were filled with enamel or dentine tissue. In places where the section cuts the tooth axially these diminutive teeth will appear with a distinct pulp canal, dentine and enamel in proportionate relations, but generally of irregular form. Many of the pulp chambers seem to be filled with calcospherites.

Heretofore it has been difficult to diagnose an odontome, but with the X-ray and sharp steel probes it ought not to be a difficult matter to distinguish and locate them, so that their removal may be easily accomplished.

### DISCUSSION.

Dr. Thos. Fillebrown, of Boston: I have known of but one case of an odontome in fifteen years, they are so rare as not to have attracted much attention. Few molar or incisor teeth are found in these odontomes, just why, we can't explain, except because of their being formed usually at the time of the development of these germs. I don't see how we can differentiate an odontome from a tumor, as we know so little as to their origin.

Dr. F. E. Logan, of Chicago: A cyst containing one tooth may be a tumor, and one containing many might be classed an odontome. A dentigerous cyst is made by encapsuling the tooth or teeth in a fibrous membrane. These cysts are difficult of diagnosis because there is no suppuration. They can be distinguished from necrosed bone by the feeling with a steel probe, and their removal should always be preceeded by an exploratory operation.

Dr. W. C. Barrett, of Buffalo: I have in my possession an odontome taken from a colt's jaw which contains nearly a quart of teeth. These odontomes are often found in the lower animals and they might be studied successfully from the standpoint of comparative anatomy. I have never seen an odontome which exceeded the arch type of dentition, or forty-eight teeth.

Dr. J. S. Marshall, of San Francisco, U .S. A.: This is a new subject and exceedingly important to bring before this society. These cases are rare. I came across one a few years ago, and upon looking up the literature I could find only a few recorded. In the lower animals they are more frequently found and often very large. I know of one taken from a colt which was six inches long. There is one in the Army Museum as Washington, presented by Dr. Finley. The dentigerous cysts are much more commou. In diagnosing them when deeply imbeded, use a steel probe with a rubber tube attached to an ear piece, this will give the sound as well as touch sense.

Dr. M. F. Finley, of Washington, D. C.: The case to which Dr. Marshall referred was taken from the upper jaw at the region of the third molar. It was pedunculated and one surface has the shape of a molar tooth.

Dr. Gilmer could not agree with the statement of Dr. Barrett that no more than the dentition of the arch type could be found in an odontome. We must always remember that there is a great difference between an odontome and a tumor attached to the root of a tooth.

